# Essential Oils Against Spoilage in Fish and Seafood: Impact on Product Quality and Future Challenges

**DOI:** 10.3390/foods13233903

**Published:** 2024-12-03

**Authors:** Matheus Barp Pierozan, Josemar Gonçalves de Oliveira Filho, Leandro Pereira Cappato, Adriano Carvalho Costa, Mariana Buranelo Egea

**Affiliations:** 1 Instituto Federal de Educação, Ciência e Tecnologia Goiano, Campus Rio Verde, Rio Verde 75901-970, GO, Brazil; matheus.pierozan@estudante.ifgoiano.edu.br (M.B.P.); leandro.cappato@ifgoiano.edu.br (L.P.C.); adriano.costa@ifgoiano.edu.br (A.C.C.); 2 Department of Mechanical and Industrial Engineering, University of Illinois at Chicago, Chicago, IL 60607, USA; josemar.gooliver@gmail.com

**Keywords:** microbial inactivation, volatile oils, preservatives, food safety, surface decontamination, antioxidant, food preservation

## Abstract

The preservation of fish and seafood represents a significant challenge for the food industry due to these products’ high susceptibility to microbial spoilage. Essential oils (EOs), classified as Generally Recognized as Safe (GRAS), have become a natural alternative to synthetic preservatives due to their antimicrobial and antioxidant properties. This review aims to analyze the specific potential of EOs in extending the shelf life of fish and seafood products, offering a natural and effective preservation solution. It provides a detailed overview of EOs applications and mechanisms, highlighting their role in controlling spoilage microorganisms while maintaining product quality. The main methods of EOs application include immersion, spraying, and pipetting, with antimicrobial effectiveness influenced by factors such as concentration, exposure time, and food characteristics like chemical composition and biofilms. Direct EOs application shows challenges that can be countered by exploring nanoemulsion technology as an effective strategy to enhance EOs stability and controlled release, maximizing their preservation impact. Additionally, coatings made from chitosan, gelatin, Farsi gum, and carrageenan, combined with EOs such as oregano, clove, and thyme have shown efficacy in preserving species like rainbow trout, mackerel, and shrimp. However, the commercial feasibility of using EOs in fish preservation depends on consumer acceptance and regulatory compliance. This review offers valuable insights for the industry and researchers by highlighting the practical applications and commercial challenges of EOs in seafood products, underscoring the importance of consumer acceptance and regulatory adherence for market viability.

## 1. Introduction

Fish and seafood preservation is a crucial concern for the food industry, as consumers demand products that are fresh and minimally processed [1]. Fresh and healthy fish are generally sterile, as the fish’s immune system prevents bacteria from growing in the flesh [2]. After the death and collection of fish or seafood during storage, microorganisms invade the meat by moving between the muscle fibers [3]. Fish and seafood are excellent culture media for microorganisms due to intrinsic and extrinsic factors, such as high nutritional composition, water content, and pH, which favor microbial growth [4,5].

Thus, the fishing industry has great difficulty in maintaining fish and seafood quality, often due to the considerable distances between consumers and harvesting or production areas, offering opportunities for microbial growth and recontamination [6].

In addition to contamination by natural microbiota, fish and seafood can be easily contaminated during processing, both by spoilage microorganisms and by important pathogens that cause foodborne outbreaks, such as *Escherichia coli*, *Salmonella* spp., *Listeria monocytogenes*, and *Staphylococcus aureus* [7]. Although these pathogens are not part of the natural microbiota of fish or seafood, they can become asymptomatic hosts, causing cross-contamination in the industrialization and marketing stages [8]. This fact occurs mainly due to handling and the great capacity for biofilm formation, representing great dangers for the food industry [9].

Transporting fish and seafood in closed systems causes changes in their metabolic state, which can result in oxidative damage due to the generation of reactive oxygen species (ROS). This increase in ROS production often culminates in lipid peroxidation, which can be quantified by the concentration of thiobarbituric acid-reactive substances (TBARSs). In addition, this process causes damage to proteins. It compromises the antioxidant defense capacity of fish and seafood, increasing vulnerability to environmental stresses and reducing the quality of the final product [10].

Among the chemical degradation processes, lipid oxidation—usually initiated by the presence of free radicals—emerges as the most prevalent cause, resulting in rancidity, discoloration, and accumulation of potentially toxic compounds, such as hydroperoxides, aldehydes such as malonaldehyde, and other toxic by-products that are harmful to human health [11,12,13,14]. Therefore, protecting these products from bacteria, fungi, and other contaminants is essential to ensure that fish and seafood are safe and of high quality. In this context, the main methods employed to extend the shelf life of meat and meat products include the suppression of lipid oxidation and the prevention of bacterial growth [15,16].

To avoid contamination, additional techniques to conventional processes have shown the potential to improve food safety and quality [17]. In this sense, food additives and preservatives create barriers to microbiological growth, extend shelf life, and improve or maintain nutritional value, providing food safety [18].

The quantity and conditions under which food additives can be used are described in the “Norma General Para Los Aditivos Alimentarios”, in which foods are divided into sixteen categories [19]. Preservatives, which correspond to a category of food additives, function as antimicrobial agents that inactivate or inhibit the growth of microorganisms in food [20].

Advances in new technologies have driven the use of natural antimicrobials, gaining preference among consumers over synthetic additives due to the potential adverse health effects of the latter [21,22,23]. The rise in microbial resistance to antibiotics and the negative impacts of synthetic additives have accelerated this transition. Consequently, consumers and the food industry are increasingly inclined to adopt natural antimicrobial agents for food preservation and disease control [24]. Growing concerns about the safety of synthetic products have led the industry to search for natural alternatives [23,25,26].

Antimicrobial agents, such as organic acids, essential oils (EOs), and bacteriocins, have been highlighted in preserving fish and seafood, extending the shelf life of these products and ensuring their safety [27]. In particular, EOs are promising due to their excellent bioactive properties, including antimicrobial, antioxidant, anti-inflammatory, and immunomodulatory actions [28]. Furthermore, EOs are considered natural, safe, and biodegradable alternatives and can reduce the emergence of antibiotic-resistant strains [29]. Due to this great potential, the global EO market size was estimated at USD 8.8 billion in 2022 and is expected to grow at a compound annual growth rate (CAGR) of 11.8% to reach a value of USD 15.3 billion by 2027 [30].

Several EOs—including citrus, mentha, origanum, thymus, zataria, and zingiberaceae—have shown significant potential in reducing microbial load in fish and seafood, helping to ensure compliance with EU safety standards for raw fish. Among these, origanum, zingiberaceae, and thymus stand out for their broad-spectrum efficacy, effectively combating many microorganisms, including anaerobic H_2_S-producing bacteria. When applied correctly, using methods such as active films or emulsions, these EOs can extend the shelf life of seafood, slowing sensory deterioration by up to five times compared to untreated fish and seafood, without significantly altering the product’s natural odor [31].

Standardizing EOs ensures reproducibility and consistency in preserving seafood and marine products. However, most regulations still do not adequately govern the use of EOs. For example, no regulatory agency certifies or approves EOs regarding their quality and purity in the United States. Variations in the composition and concentration of essential oils can lead to inconsistent antimicrobial and sensory effects, compromising preservation effectiveness. Therefore, it is essential to establish clear guidelines and protocols for using EOs in fish and seafood preservation, ensuring effective and reliable methods to preserve these valuable food resources [32].

To effectively incorporate EOs into fish and seafood products, it is essential to deeply understand their mechanisms of action, appropriate concentrations, and optimization of delivery systems to overcome their natural limitations. Therefore, this review aims to present a comprehensive overview of the use of EOs in fish and seafood, highlighting them as a natural alternative in combating microorganisms, thus aiming to extend the shelf life of these products.

## 2. Essential Oils (EOs): An Overview

The European Chemicals Agency (ECHA) defines EOs as the volatile part of a natural product, which can be obtained by distillation, steam distillation, or, in the case of citrus fruits, pressure distillation [33]. The oil is “essential” because it has the plant’s distinctive aroma or essence. The International Organization for Standardization (ISO) defines an EO as a product obtained from a natural raw material of plant origin by steam distillation, by mechanical processes from the epicarp of citrus fruits, or by dry distillation after separation of the aqueous phase, if there is any, by physical processes [34].

The production of EOs in plants involves complex metabolic pathways centered mainly on the biosynthesis of terpenoids, which are the main constituents of these oils. The biosynthesis of EOs in aromatic plants is facilitated by two main pathways: the mevalonic acid (MVA) pathway and the 1-deoxy-d-xylulose-5-phosphate (DXP) or 2-C-methylerythritol-4-phosphate (MEP) pathway. These pathways produce the universal precursors iso-pentenyl diphosphate (IPP) and dimethylallyl diphosphate (DMAPP), which are subsequently converted to prenyl diphosphates by prenyl diphosphate synthases. These compounds serve as substrates for the synthesis of geranyl diphosphate (GPP) and farnesyl diphosphate (FPP), which are crucial intermediates in the formation of terpenoids by terpenoid synthases (TPS) [35].

**Figure 1:**
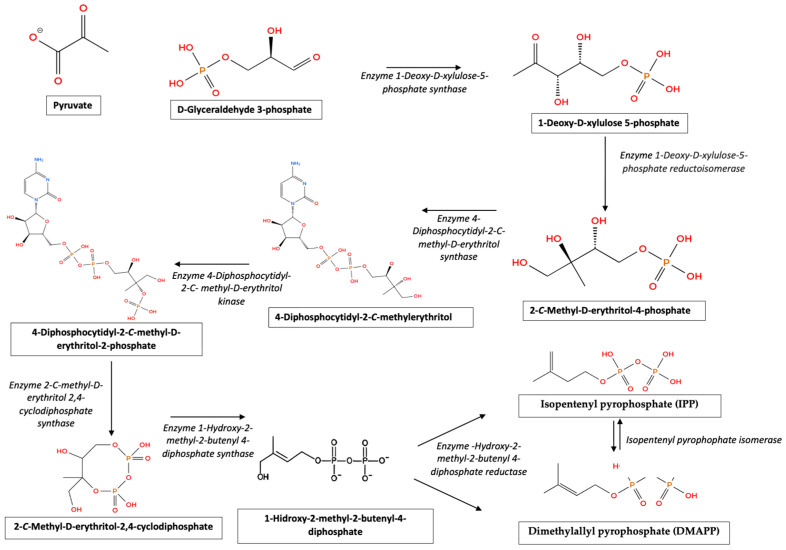
The mevalonate (MVA) pathway. The functional groups are highlighted in red to facilitate identification and visualization.

The MVA pathway (Figure 1), located in the cytosol, mainly leads to the formation of sesquiterpenes. In contrast, the MEP pathway, which occurs in plastids, synthesizes mono- and diterpenes. In addition, the shikimic acid pathway contributes to the formation of phenylpropenes, another class of compounds found in EOs [36]. These pathways are not linear but involve complex networks with multiple branching points, allowing significant chemical diversification and the formation of various terpenoid structures [37].

Recent advances in metabolic engineering have opened new avenues for optimizing EO production. This involves reconstituting biosynthetic pathways in heterologous organisms, such as microorganisms, through synthetic biology. This approach allows for the cloning and expression of multiple genes, enabling EO production in non-native hosts, which may be more efficient and sustainable than traditional plant extraction methods [38].

Volatile organic compounds synthesized by the secondary metabolism of plants serve as attractants for species pollinators, while volatiles emitted by vegetative parts, especially those released after herbivory, appear to protect plants by deterring herbivores and attracting enemies of herbivores [39]. Furthermore, they are produced in response to physiological stress, pathogen attack, and ecological factors, playing an important role in plant defense, as they often have antimicrobial properties [40]. Several compounds form these EOs, essentially volatile hydrocarbons in plants, such as terpenes, terpenoids, fatty acid derivatives, aromatic components derived from phenol, and aliphatic components [41].

EOs can be classified in several ways, depending on the specific oils being considered. Each classification type helps to understand essential oils’ properties, uses, and characteristics better. However, the most common classifications are based on the aromatic notes they emit, the botanical families their source plants belong to, their therapeutic properties based on the extraction method, and their chemical composition [42,43].

The components of essential oils can be chemically classified based on four main criteria: (1) the primary biosynthetic origin; (2) the size or number of carbon atoms; (3) the fundamental structure or “skeleton” of the molecule; and (4) the electron-withdrawing carbon atoms, such as oxygen, nitrogen, or sulfur, which are elements larger than carbon [44].

**Figure 2:**
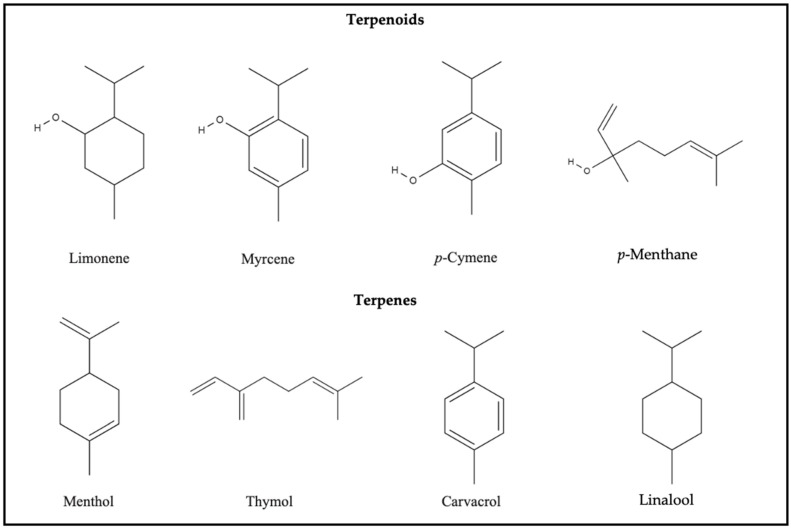
Structural chemical formulas of some terpenes and terpenoids.

Terpenes or isoprenoids belong to the largest class of secondary metabolites and consist of five carbon isoprene units that are assembled (many isoprene units, C_5_H_8_) in thousands of ways (Figure 2). Terpenes are simple hydrocarbons. At the same time, terpenoids are a modified class of terpenes with different functional groups and oxidized methyl groups moved or removed at various positions. Depending on their carbon units, terpenoids are divided into monoterpenes, diterpenes, sesquiterpenes, and triterpenes, depending on their carbon units [37]. Further, terpenoids can be divided into alcohols, aldehydes, esters, ethers, epoxides, ketones, and phenols. Examples of terpenoids are carvacrol, citronellal, geraniol, linalool, linalyl acetate, piperitone, menthol, and thymol, with the antimicrobial activity of most terpenoids being linked to their functional groups [45].

EOs are generally liquids at room temperature, volatile, clear, and rarely colored, lipid-soluble and soluble in organic solvents with a density generally lower than the density of water, soluble in alcohol, ether, and fixed oils, but insoluble in water, and have a very high refractive index and optical activity [46].

The use of EOs in food intended for human consumption is governed by a complex set of international regulations, each with specific guidelines to ensure product safety and quality. These regulations vary by jurisdiction, reflecting public health and food integrity concerns. The FDA recognizes a significant number of EOs as GRAS (Generally Recognized As Safe) substances for human consumption [47]. EOs, oleoresins (solventless), and natural extractives (including distillates) that are generally recognized as safe for their intended use are listed by the Code of Federal Regulations (CFR) 21 CFR 182.20 [48].

US federal regulations for EOs are based on the application category: antimicrobial agents (21 CFR § 170.3(o)(2)), Food Additive (21 CFR 170.3(e)(1)), Flavor enhancers (21 CFR 170.3(o)(11)), Flavoring agents and adjuvants (21 CFR 170.3(o)(12)), Food contact substance (21 CFR 170.3(e)(3), Natural flavor or natural flavoring (21 CFR 101.22(a)(3)), Preservatives (21 CFR 172.5), and Processing aids (21 CFR 101.3(ii)) [49].

EOs are considered safe for consumers. However, their potential toxicity should not be overlooked. Although they are promising in food safety, their consumer safety depends on several factors, such as the type of EO, concentration, and individual sensitivities. Therefore, several laws are being reviewed to understand advances in using EOs [50].

## 3. EOs with Antimicrobial Properties: Mechanism of Action

EOs have emerged as promising natural antimicrobial agents in the food industry, offering an alternative to synthetic preservatives. Their efficacy against foodborne pathogens, such as bacteria and fungi, increases food safety and extends product shelf life [51].

The antimicrobial activity of EOs is often associated with their lipophilic character and lower molecular weight, which facilitate penetration into cell membranes of the monoterpenes present in them. These lipophilic compounds allow EOs to interact with the lipid layer of bacterial and mitochondrial cell membranes, making them more permeable. This affects pH homeostasis and causes the leakage of inorganic ions and other cellular contents, such as protons, phosphates, and potassium [52,53,54]. This interaction can cause the loss of electron-dense cellular material, coagulation of cytoplasmic constituents, and cell wall rupture, leading to cell death [55,56,57,58].

EOs have demonstrated greater bacteriostatic activity against Gram-positive bacteria and direct antibacterial activity against Gram-negative bacteria [59]. This is due to the difference in the peptidoglycan layer in Gram-positive bacteria, which is exposed outside the outer membrane. Li et al. [51] used electron microscopy in Gram-positive bacteria to demonstrate that tea tree oil (*Melaleuca alternifolia*) caused plasmolysis and partial disruption of the cytoplasmic membrane in *E. coli* and *S. aureus* strains. Furthermore, EO from *Origanum vulgare* L. caused rapid loss of absorbent material and release of potassium ions in *S. aureus*, indicating that the accumulation of EO compounds compromises the integrity of the cell membrane [60,61]. Meanwhile, in Gram-negative bacteria, an outer membrane comprises a bilayer of phospholipids linked to the inner membrane by lipopolysaccharides (LPSs). LPSs, which include lipid A, a central polysaccharide, and the O side chain, confer additional resistance to Gram-negative bacteria against EOs [62].

Furthermore, the efficacy of EOs is dose-dependent. For example, Galgano et al. [63] observed more pronounced bactericidal effects in Gram-positive bacteria and a dose-dependent effect in Gram-negative bacteria. Another strategy to increase the efficacy of EOs is the combination of different components. Combining the components of several EOs, such as carvacrol and thymol, can synergistically increase antimicrobial activity. Adding small amounts of these compounds increases cell permeability and causes ion leakage, compromising homeostasis and ionic balance [52]. Studies also indicate that when used alone or in combination, phenolic antimicrobials present non-linear dose responses, highlighting the importance of adjusting concentrations and combinations to optimize the antimicrobial effect [64].

A challenge regarding using EOs is the lack of standardization in methodologies for evaluating the antimicrobial activity of EOs. Variability in methods, such as agar amounts, EO volumes, and minimum inhibitory concentration (MIC) definitions, makes direct comparison of results difficult. Standardization in test methods is essential to obtain a more consistent and reliable evaluation [63,65].

The change in the chemical composition of essential oils due to extrinsic factors such as cultivation method, geographic location, harvest time, and amount of water and nutrients, among others; and intrinsic to the plant, such as plant age and genetics, and stage of vegetative development [59], is also considered a challenge in EO use. In the work of Sarrou, Martens, and Chatzopoulou [66], two cultivated varieties of *Salvia fruticosa* Miller, Lamiaceae (syn. *S. triloba* L.), were investigated and evaluated for their EOs, phenolic composition, and antioxidant activity during different harvest seasons. The qualitative profiles of the two populations were similar, while significant differences were observed in the quantitative composition under different harvest times. An increase in the content of the EO and its major compound, 1,8-cineole, was observed from spring to summer (April–August), and a decrease was observed in autumn (September–October).

This variable chemical quality presents an issue in the use of EOs since the biological effects of their use depend on the chemical composition, that must be elucidated. This variability affects the reproducibility of their biological activity since even small changes in the composition can alter their effectiveness against microorganisms and their antioxidant capacity [67]. 

## 4. Methods of Applying Pure EOs in the Preservation and Quality of Fish and Seafood

**Figure 3:**
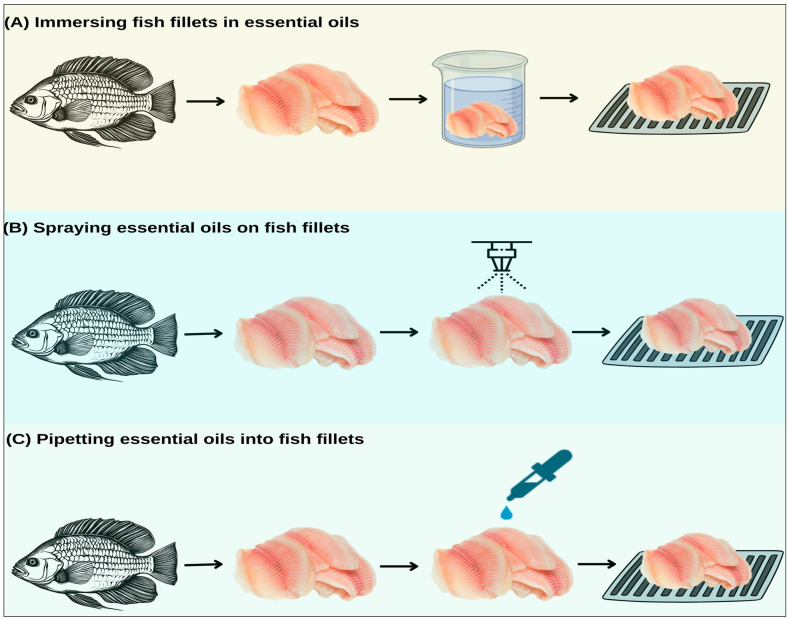
Scheme for applying essential oils (EOs) by immersion (**A**), spraying (**B**), and pipetting (**C**) in fish and seafood.

Different methods can be used to apply EOs in fish and seafood preservation, and the choice of method can directly influence the efficacy of these compounds. In addition, factors related to the EO, such as its concentration, exposure time, and administration method, and those related to the nature of the fish or seafood, including its chemical composition, texture, and presence of biofilms, also play a crucial role in determining the efficacy of EOs [31]. Figure 3 presents the main methods of application of pure EOs in fish and seafood described in the literature, including immersion (Figure 3A), spraying (Figure 3B), and pipetting (Figure 3C).

### 4.1. Immersion of Fish and Seafood in EOs

One of the most common methods for applying EOs to fish and seafood is immersion (Figure 3A), followed by draining the product (Table 1). This technique ensures rapid application and uniform distribution of the EO on the surface of the fish, maximizing the antimicrobial effect and prolonging the products’ shelf life and microbiological quality [68,69]. However, in the immersion process, the use of emulsifiers such as Tween 80—one of the most widely used emulsifiers—is crucial for dispersing essential oils (EOs) in aqueous solutions, particularly in food and pharmaceutical applications. These substances help create stable emulsions, enhancing the bioavailability and stability of bioactive compounds. Tween 80 reduces the interfacial tension between immiscible phases, preventing emulsion separation. While Tween 80 is commonly used, Tween 20 can also be effective in certain formulations, such as in the nanoemulsion of cinnamon essential oil [70].

Karoui and Hassoun [71] observed that immersion treatments with rosemary and basil EOs increased the shelf life of mackerel fillets by 2 and 5 days, improving the product’s color and texture.

Several species, such as cod (*Gadus morhua*), sardines (*Sardina pilchardus*), rainbow trout (*Oncorhynchus mykiss*), tiger shrimp (*Penaeus monodon*), and salmon (*Salmo salar*), were treated with EO solutions, using the immersion technique to improve conservation and sensory quality (Table 1).

In a study by Desai et al. [72] using the immersion technique for thyme, oregano, and carvacrol EOs, the authors demonstrated strong antimicrobial activity against *L. monocytogenes* in fresh catfish fillets. These EOs and carvacrol were solubilized by diluting them (1:1) in propylene glycol, and the desired concentrations were then prepared in sterile deionized water or TSB. Concentrations of 0.5% efficiently reduced 4 log CFU/mL in 30 min, while concentrations of 0.1% required more than 24 h for the same effect. On the other hand, lemon, orange, and tangerine EOs at 0.5% maintained the initial load of 4 log CFU/mL unchanged for 10 days at 4 °C, with concentrations of 1% being bacteriogenic over time.

Another study by Sadeghzadeh, Javadi, and Mogaddam [73] showed that higher concentrations of EOs (2000 mg/L of *Ziziphora tenuior* L. or *Artemisia dracunculus*) in rainbow trout fillets significantly reduced volatile nitrogenous bases (TVB-N) and microbial counts, especially 2000 mg/L of *Z. tenuior*, which was more effective in controlling spoilage than *A. dracunculus*.

Lambrianidi et al. [74] evaluated the effects of chitosan and oregano EO on preserving refrigerated and vacuum-packed European eel fillets and assessed the microbiological, chemical, and sensory changes. The treatments with chitosan and oregano EO by immersion were shown to inhibit bacterial growth, prolonging the shelf life of the fillets. The samples treated with chitosan and the combination of chitosan with oregano oil showed the most extended durability, reaching 18 days of shelf life, while the samples treated only with oregano oil lasted 14 days, compared to 10 days for the control group. In the sensory evaluation, the samples treated with chitosan stood out, receiving the best evaluations due to the citrus odor, while the samples treated with oregano oil presented a slight bitterness and metallic taste. Combining chitosan with oregano oil helped to tone down these undesirable flavors.

Common carp (*Cyprinus carpio*) fillets were immersed in a 0.1% cinnamon essential oil (EO) solution, prepared by dissolving 1 g of cinnamon EO in 1 L of sterile water. To aid in the even distribution and incorporation of the cinnamon EO, 0.2 g of Tween 80 was added to the solution. The fillets were then vacuum-packed and stored at 4 °C. Using high-throughput sequencing, 290,753 bacterial sequences and 162 genera were identified, revealing significant microbial diversity. The cinnamon EO treatment reduced the presence of spoilage bacteria, particularly *Aeromonas* and *Lactococcus*, extending the shelf life of the fillets by approximately 2 days. Furthermore, the treatment inhibited the increase in TVB-N and biogenic amines, such as putrescine and cadaverine, thereby improving sensory quality [66].

The sanitization immersion method, although effective, has significant limitations, such as the risk of bacterial cross-contamination in the sanitizing solution and the high use of sanitizer compared to more efficient methods, such as nebulization, which use less volume and improve the distribution of the disinfectant [75,76].

### 4.2. Spraying Fish and Seafood with EOs

Spraying (Figure 3B) is a common method of applying EOs in the food industry due to its simplicity, speed, and good surface coverage. It allows for uniform distribution of EOs across the product surface without saturating the solution, as can occur with immersion. However, concerns include the possible loss of volatile EO compounds during spraying and the need for additional precautions to protect workers from exposure to these compounds [77,78].

An example of its efficacy is the spraying of bay laurel (*Laurus nobilis*, 2.0%) EO combined with vacuum packaging for rainbow trout (*Oncorhynchus mykiss*) fillets, which resulted in significantly lower values of TVB-N, pH, and bacterial counts (*Enterobacteriaceae*, *Pseudomonas* spp., psychrotrophic, and total bacteria) compared to the control group during refrigerated storage. The treated fillets showed better sensory scores, indicating superior quality and freshness, and shelf life was extended by about 4 days [79].

Furthermore, Motavaf et al. [80] showed that spraying *Zataria multiflora* EOs (0.5%, 0.8%, and 1.5% (*w*/*w*)) combined with potassium sorbate (2%) on rainbow trout fillets was effective in controlling *L. monocytogenes* PTCC 1.163. The treatment with 1.5% *Zataria multiflora* EO achieved the greatest reduction in *Pseudomonas aeruginosa*, total mesophilic aerobic bacteria count, TBARSs, and TVB-N, in addition to presenting the best sensory scores over 12 days. Gas chromatography and mass spectrometry identified carvacrol (66.2%) and thymol (26.5%) as the main components responsible for the observed antibacterial activity.

Furthermore, one of the advantages of spraying is the possibility of applying edible coatings, with the ability to create a uniform layer with precise thickness control and the ease of applying multiple layers, allowing greater flexibility in the composition of the coating. In addition, the spraying process minimizes contamination of the coating solution and allows temperature control, making it ideal for industrial-scale automation. However, spraying also has disadvantages, such as the requirements to adjust the pressure to avoid destroying the film structure and ensure homogeneous contact between the drops to form a continuous coating. In addition, the system requires higher maintenance and operating costs when compared to other methods [81].

### 4.3. Pipetting of Fish and Seafood with EOs

Pipetting (Figure 3C) is another method used for applying EOs, offering a precise and controlled application of antimicrobial and antioxidant compounds, and is widely used in scientific research at the laboratory level [82]. This method allows for the accurate measurement of the application of EOs and their controlled incorporation into the food matrix, which is crucial in fish and seafood, where the efficacy of EOs can be affected by high levels of lipids and proteins. Pipetting also allows for targeted application, minimizing the required amount and potentially reducing the impact on the product’s sensory properties [83].

Pyrgotou et al. [84] evaluated the pipetting of oregano (*Thymus vulgaris*) EO followed by massaging for uniform distribution of the EO in salted rainbow trout fillets packed in a modified atmosphere (45% CO_2_, 5% O_2_, and 50% N_2_) and stored at 4 °C for 21 days. Concentrations of 0.2% and 0.4% oregano EO were tested, in addition to a control group. EO treatments significantly reduced microbial counts of lactic acid bacteria (LAB), H_2_S-producing bacteria, *Enterobacteriaceae*, and *Pseudomonas* spp., as well as TVB-N and TMA-N values. Treatment with 0.2% oregano oil extended the shelf life of the fillets by 7–8 days, while treatment with 0.4% was considered inadequate due to the strong odor.

In the work of Tosun et al. [85], the pipetting of EOs from coriander, garlic, rosemary, and orange peel was evaluated on the survival of *S. enteritidis* and *L. monocytogenes* in fresh Atlantic salmon stored at 2 °C. The results showed a significant reduction in the population of both bacteria compared to the control group. Coriander EO provided the greatest reduction in the population of *S. enteritidis*, followed by garlic, orange peel, and rosemary EOs. Coriander and orange peel EOs also effectively reduced the populations of *L. monocytogenes.*

However, while pipetting of EOs in fish has proven advantageous in laboratory studies, allowing precise and controlled application of antimicrobial and antioxidant compounds with reduced sensory impact, the technique is limited on an industrial scale due to uneven distribution, low efficiency, and the need for large volumes and manual operations even for standardization, which compromise the homogeneity of the product. In addition, manual errors can accumulate in multi-step processes, compromising the consistency of the procedure and the time involved in the process [86].

Unlike immersion, which allows direct and uniform contact of EOs across the entire surface of the submerged product, pipetting is a technique that applies EOs to specific points of the sample. Due to this localized application, it is essential to ensure the substances spread properly to achieve uniform distribution. This is usually accomplished through techniques such as massaging, which help disperse the EOs across the product’s surface. However, the technique requires greater care to avoid uneven distribution, which could compromise the antimicrobial or antioxidant efficacy of the EOs. Therefore, the success of pipetting depends not only on the application method but also on the properties of the EO used, such as its viscosity and spreading ability, to ensure homogeneity and efficient dispersion of the EOs.

**Table 1:** Fish and seafood products that received the application of essential oils (EOs) through immersion, spraying, pipetting, direct mixing in processed products, and vaporizing.

Fish and Seafood Type	EOs	Application	Application Details	Main Results	Reference
Cod (*Gadus morhua*) from the Baltic Sea and salmon (*Salmo salar*)	Basil (*Ocimum basilicum* L.), bay *(Laurus nobilis*), clove (*Syzygium aromaticum* L.), lemongrass (*Cymbopogon citratus*), marjoram (*Origanum majorana* L.), sage (*Salvia officinalis*), thyme (*Thymus vulgaris*), oregano (*Origanum vulgare*), and cinnamon (*Cinnamomum zeylanicum*) EOs	Immersion	Immersion of fillets in sterile deionized water solution containing treatments with each EO separately and packaged in a modified atmosphere and stored at 2 °C.	Oregano EO extended the shelf life of contaminated cod fillets from 11 to 12 days to 21–26 days at 2 °C.	[87]
Bluefish (*Pomatomus saltatrix*)	Thyme (*Thymus vulgaris*) and laurel (*Laurus nobilis*) EOs	Immersion	Homogenized with 1% EO volume per fish flesh weight (*v*/*w*) of thyme EO or 1% EO volume per fish flesh weight (*v*/*w*) of laurel EO.	Thyme EO was most effective in reducing lipid oxidation, followed by bay oil, extending the shelf life of anchovies by 3–4 days during storage on ice for 13 days.	[88]
Raw catfish	Thyme (*Thymus vulgaris*), carvacrol, lemon (*Citrus limon*), orange (*Citrus sinensis*), oregano (*Origanum vulgare*), and tangerine (*Citrus reticulata*) EOs	Immersion	Immersion of fillets for 30 min in EO solutions followed by storage at 4 °C for up to 10 days.	Immersion in carvacrol EO reduced *L. monocytogenes* to an undetectable level from its initial load of 5 log CFU/g and reduced the total microbial load of catfish fillets by approximately 5 log CFU/g. A negative sensory implication occurred in the sensory analyses on samples treated with carvacrol.	[72]
Hepatopancreas of Pacific white shrimp (*Litopenaeus vannamei*)	Lemon (*Citrus limon)*, plai (*Zingiber cassumunar* Roxb) and basil (not cited) EOs	Immersion	Addition of EOs by immersion followed by storage for 10 days at 30 °C.	Lemon EO helped to reduce the formation of volatile compounds and rancid odor.	[89]
Sardines (*Sardina pilchardus*)	Lemon (*Citrus limonum* L.) and bergamot (*Citrus aurantium* L.) EOs from Algeria	Immersion	The sardines were placed in plastic bags, followed by the addition of EO, and stored in refrigeration (8 ± 1 °C) until the 7th day.	Bergamot EO, when applied at 4 times the minimum inhibitory concentration, completely stopped the growth of *S. aureus* from the second day of storage.	[90]
Salmon (*Salmo salar*) and scampi (*Penaeus monodon*)	Oregano (*Oreganum compactum*), cinnamon (*Cinnamomum zeylanicum*), and thyme (*Thymus zygis* ct. Thymol) EOs	Immersion	Marinate for 2 min and store at 4 °C.	In shrimp, LAB growth was reduced for at least six days with oregano and thyme EOs.	[70]
Atlantic Mackerel (*Scomber scombrus*)	Rosemary (*Rosmarinus officinalis*) and Basil (*Ocimum basilicum* L.) EOs	Immersion	Product immersion in EOs for 30 min at 2 °C. Samples were packaged in airtight polyamide/polyethylene (PA/PE 90) packaging under atmospheric conditions, sealed, and stored at 2 °C for 13 days.	EO basil extended the shelf life of Atlantic mackerel fillets by 2 days, while EO rosemary extended it by 5 days compared to the control group.	[71]
Common carp (*Cyprinus carpio*)	Cinnamon (*Cinnamomum cassia* Presl) EO	Immersion	Fillets were immersed in EO solutions for 10 min. Then, the fillets were vacuum packed in polyethylene/polyamide film bags and kept refrigerated (4 ± 1 °C) for 14 days.	Cinnamon EO treatment maintained good quality and extended the shelf life of vacuum-packed carp fillets by 2 days.	[91]
Grass carp (*Ctenopharyngodon idellus*)	Cinnamon bark (*Cinnamomum tamala*) EO	Immersion	The fillets were immersed in EO emulsion for 30 min, stored in polyvinyl chloride bags, and refrigerated (4 ± 1 °C).	Improved sensory quality by inhibiting microbial growth and slowing the increase in TVB-N, putrescine, and cadaverine concentrations and K value.	[92]
Cod (*Gadus morhua*)	Citral, carvacrol, thymol, and eugenol EOs	Immersion	Portions of cod were immersed for 30 s in the treatment solutions, drained for 15 s, exsanguinated in sterile distilled water for 30 s, and stored under refrigeration at 2 °C for 18 days.	The treatments were not successful in inhibiting growth and extending the lifespan of the cod.	[93]
Sous-vide sea bass (*Dicentrarchus labrax*)	Laurel (*Laurus nobilis*) and basil (*Ocimum basilicum*) EOs	Immersion	Marination for 1 to 4 h, followed by placing in a water bath at 80 °C, cooking until the core temperature reaches 70 °C, and storage at 4 °C for 15 days.	The fillets incorporated with basil EO exhibited the lowest TBARS values on the last day and on day 3 of storage, indicating better inhibition of lipid oxidation compared to bay leaf EO and control samples.	[94]
Rainbow trout	Tarragon (*Artemisia dracunculus*) and *Ziziphora tenuior* L. (Kakuti in Persian) EOs	Immersion	Immersion in oils for 10 min and storage at 4 °C for 15 days.	*Z. tenuior* EO was more effective than *Artemisia dracunculus* EO in controlling microbial load, and extending shelf life of rainbow trout fillets at refrigeration temperatures.	[73]
Rainbow trout (*Oncorhynchus mykiss*)	Laurel (*Laurus nobilis*) EO and vacuum packaging	Spraying	The application was performed by spraying, followed by storage at 4 °C for 14 days.	The addition of bay EO to the fillets extended the shelf life by approximately 4 days and improved the sensory characteristics.	[79]
Rainbow trout	*Zataria multiflora* EO and potassium sorbate	Spraying	Application by spraying, followed by packaging in a modified atmosphere (30% N_2_, 30% O_2_, and 40% CO_2_), and storage at 4 ± 1 °C for 12 days.	The use of *Z. multiflora* EO in rainbow trout fillets was most effective in reducing the growth of inoculated *L. monocytogenes*, total bacterial counts, TVB-N, TBA, and *P. aeruginosa* counts. Sensory attributes, including odor, flavor, and texture, were better preserved in fillets treated with *Z. multiflora* EO.	[80]
Asian sea bass (*Lates calcarifer*) fish	Thyme (*Thymus vulgaris*) and oregano (*Origanum vulgare*) EOs	Pipetting	Packaging of fish in polyethylene plastic bags; addition of EOs on the side of the bags with a micropipette; storage under refrigeration between 0 and 2 °C for 33 days.	Sensory analysis showed that fish treated with EOs presented better quality attributes than the control group, with significant differences after 12 days of storage. Fish treated with EOs had significantly lower bacterial counts, with no bacterial growth detected, while fish in the control group showed increased bacterial counts.	[95]
Rainbow trout (*Oncorhynchus mykiss*)	Oregano (*Thymus vulgaris*) EO and modified atmosphere packaging (45% CO_2_/5% O_2_/50% N_2_)	Pipetting	Application of EO on fresh salted fillets with micropipette, uniform distribution of EO and storage under refrigeration at 4 °C for 21 days.	Oregano EO resulted in a 7-to-8-day shelf-life extension for fresh trout fillets.	[84]
Sea bream (*Sparus aurata*)	Laurel (*Laurus nobilis*) and cumin (*Cuminum cyminum*) EOs	Pipetting	Application of EOs on fillets with micropipette, uniform distribution of EO, vacuum packaging in polyethylene/polyamide bags and stored in the refrigerator (2–4 °C) for 20 days.	EOs reduced bacterial growth by approximately 0.5 to 1 log CFU/g and lipid oxidation by about 40% in TBA value, extending the shelf life of fish by about 5 days of ice storage. However, EO application also increased endogenous protease activation, increasing muscle proteolysis.	[96]
Atlantic salmons (*Salmo salar*)	Coriander (*Coriandrum sativum* L.), garlic (*Allium sativum*), rosemary (*Rosmarinus officinalis*), and orange peel (*Citrus sinensis*) EOs	Pipetting	Using a micropipette, EOs were applied to both sides of each fish. The samples were placed in sterile plastic bags and stored at 2 ± 1 °C for 96 h.	Coriander EO showed the greatest reduction in the *S. enteritidis* population compared to the control group, followed by garlic, orange peel, and rosemary EOs. EOs, especially coriander and orange peel, also effectively reduced *L. monocytogenes*.	[85]
Rainbow trout (*Oncorhynchus mykiss*)	Rosemary (*Rosmarinus officinalis*) EO	Mixing EO with the burger dough	The oil was added to the hamburgers, packaged in plastic film, and stored in a refrigerated room (4 ± 1 °C) for 9 days.	Lower biogenic amine content reduces putrescine, cadaverine, tyramine, and histamine levels.	[97]
Red drum (*Sciaenops ocellatus*)	Clove (*Syzygium aromaticum*), cumin (*Cuminum cyminum*), and spearmint (*Mentha spicata*) EOs	Vaporization	The fillets were placed in a container containing filter paper impregnated with oils, steamed for 2 h, packed in polyethylene bags and stored in refrigerated chambers at 4 ± 1 °C for 20 days.	It delayed the sensory deterioration of fish, exhibiting a positive effect and causing low biogenic amine content, especially histamine, putrescine, and cadaverine.	[98]

TVB-N: total volatile basic nitrogen; LAB: lactic acid bacteria; TBARSs: thiobarbituric acid-reactive substances.

### 4.4. Nanoemulsion of EOs in Fish and Seafood

Due to the aromaticity of EOs, increasing their concentration in fish and seafood can be challenging. Increasing the concentration to compensate for losses due to interaction with the product and volatility during storage can result in undesirable flavors, leading to consumer rejection [99]. Furthermore, incorporating pure EOs into fish and seafood matrices presents inferior results compared to in vitro preservation tests [100].

Nanotechnology has emerged as a promising field in food science and technology, especially in the fish and seafood sector. Due to the small size of nanoparticles, these structures present unique properties that can be innovatively exploited in food systems [101,102,103]. Nanoemulsions, kinetically stable colloidal dispersions with droplets between 20 and 200 nanometers, have gained prominence in the food industry. These structures offer advantages such as increased solubility of lipophilic compounds, protection against oxidation, and reduction in sensory alterations [104,105]. These characteristics are especially relevant for the preservation of fish and seafood products.

Techniques such as high-pressure homogenization, microfluidization, or sonication are used to prepare nanoemulsions. Generally, to prepare nanoemulsions using high-energy techniques, a premixed emulsion is subjected to a second homogenization procedure, in which large amounts of energy are applied to reduce the droplet size to the nanoscale. The final droplet size obtained will depend on the type of homogenizer, operating conditions (e.g., number of passes, pressure, etc.), emulsion composition (type of oil, surfactant, presence of biopolymers in the aqueous phase), and the physicochemical properties of the emulsion components (interfacial tension, viscosity, etc.) [106].

The application of EO nanoemulsions in fish and seafood products has shown promising results in improving the quality and extending the shelf life of these products (Table 2). They can protect EO compounds from degradation, increase their solubility, and allow controlled release, which increases the bioavailability and efficacy of the compounds [107,108].

The conversion of compounds into nanoemulsions enhances the effectiveness of EOs but also allows for a lower and more sustainable dosage for industrial applications. Regarding antioxidant activity, nanoemulsions have demonstrated inhibition of the DPPH radical by over 45%, a significantly superior result compared to non-nanoemulsified versions. This increase in antioxidant capacity occurs because the smaller droplet size increases the surface area in contact with free radicals, facilitating electron transfer and enhancing the neutralization of these radicals. Regarding antibacterial activity, nanoemulsions also stand out, showing minimum inhibitory concentrations (MICs) up to four times lower, especially against foodborne pathogens such as *Escherichia coli* and *Salmonella enteritidis*. Additionally, in terms of antibiofilm activity, nanoemulsions at concentrations up to four times lower than non-nanoemulsified versions managed to inhibit bacterial adhesion by over 67.2%. This indicates the superior effectiveness of nanoemulsions in preventing biofilm formation, a critical factor in ensuring food safety in industrial applications [109].

Furthermore, studies demonstrate that immersion of seafood fillets (such as rainbow trout and sea bass) in solutions containing nanoemulsions of EOs (such as rosemary, laurel, thyme, sage, orange, grapefruit, mandarin, lemon, mandarin, *Ziziphora clinopodioides*, and rosehip) results in significant improvements in sensory quality and shelf-life extension.

A study by Ozogul et al. [110] investigated the antimicrobial and antioxidant effects of rosemary, bay leaf, thyme, and sage EO nanoemulsions on preserving ice-stored rainbow trout fillets. The nanoemulsions showed droplet sizes ranging from 59.48 nm (sage EO) to 112.82 nm (thyme EO), with rosemary and thyme EOs being the most effective in controlling bacterial growth and reducing biochemical parameters. Immersion of the fillets in these nanoemulsions extended their shelf life to 17 days, compared to 14 days in the control samples. It also provided significant improvements in sensory, chemical, and microbiological quality. However, rosemary EO imparted a slightly bitter taste to the fillets [110].

Cinnamon EO nanoemulsions are a promising technology to enhance the antimicrobial and antioxidant properties of cinnamon EO, mainly due to the encapsulation of its active component, cinnamaldehyde, which is known for its potent antimicrobial effects against a wide range of bacteria. However, cinnamaldehyde exhibits hydrophobicity and susceptibility to oxidation, which limits the direct application of cinnamon EO in food [111,112]. Chuesiang, Sanguandeekul, and Siripatrawan [113] investigated the use of cinnamon EO nanoemulsion, produced by the phase inversion temperature (PIT) method, as a natural preservative to extend the shelf life of chilled Asian sea bass fillets. The research compared different concentrations of the nanoemulsion with bulk cinnamon EO and sodium hypochlorite, demonstrating that the nanoemulsion was more effective, with a mean droplet diameter of approximately 50.71 nm. It inhibited bacterial growth by 1–3 log CFU/g, reduced the increase in TVB-N, and retarded lipid oxidation while maintaining the sensory quality of the fillets. Nanoemulsion treatment extended the shelf life of the fillets to 6–8 days, compared with 2–4 days for the control samples, highlighting its potential as a natural preservative for the seafood industry.

Thyme EO nanoemulsions were used to preserve rainbow trout fillets during 9 days of refrigerated storage. The nanoemulsions, with diameters of 219 nm and 163 nm, demonstrated high efficacy in reducing the growth of total mesophilic and psychrotrophic aerobic bacteria, resulting in a significant decrease in microbial counts compared to control samples. Treatment with thyme oil nanoemulsions reduced total mesophilic aerobic bacteria from 6.42 to 4.62 log CFU/g and limited the growth of psychrotrophic bacteria by 1.51 log CFU/g [114].

Therefore, applying EO nanoemulsions in preserving fish and seafood proves to be an innovative and effective strategy to extend the shelf life and improve the quality of these products. This technology provides a protective barrier that preserves the active compounds of essential oils, enhancing their antimicrobial and antioxidant properties without compromising the flavor or texture of the food.

**Table 2:** Application of essential oils (EOs) through nanoemulsions in fish and seafood products.

Fish and Seafood Type	Nanoemulsion Type	Application	Main Results	Reference
Rainbow trout (*Oncorhynchus mykiss*)	Nanoemulsions containing herb oils rosemary (*Rosmarinus officinalis*), laurel (*Laurus nobilis*), thyme (*Thymus vulgaris*), and sage (*Salvia officinalis*)	The fillets were immersed for 3 min in EO nanoemulsions, wrapped in permeable stretch film, and stored in Styrofoam boxes with ice at 2 ± 2 °C for 24 days.	Nanoemulsions improved the sensory quality of rainbow trout fillets. Extended shelf life to 17 days compared to 14 days in the control group.	[110]
Rainbow trout (*Oncorhynchus mykiss*)	Nanoemulsions of orange (*Citrus sinensis*), grapefruit (*Citrus paradisi*), mandarin (*Citrus reticulata* L.), and lemon (*Citrus limon*) EOs	The fish fillets were immersed for 3 min in nanoemulsions prepared with different EOs (orange, grapefruit, mandarin, and lemon), and then stored at 4 ± 2 °C for 16 days.	The shelf life of rainbow trout fillets was extended by 2 days with the Tween 80 treatment, 4 days with the orange and lemon EO treatment, and 6 days with the tangerine and grapefruit EO treatment compared to the control group.	[115]
Rainbow trout (*Oncorhynchus mykiss*)	Orange (*Citrus sinensis*), lemon (*Citrus limon*), mandarin (*Citrus reticulata* L.), and grapefruit (*Citrus paradisi*) EOs	The fish fillets were immersed in nanoemulsions prepared with different EOs for 3 min, and then stored in a refrigerator (4 ± 2 °C) for 16 days.	The orange and lemon nanoemulsion groups effectively reduced histamine formation in the fillets.	[116]
*Oncorhynchus mykiss*	Nanochitosan and *Ziziphora clinopodioides* EO	The fillets were wrapped in films containing different concentrations of nanochitosan and *Ziziphora clinopodioides* EO and stored under refrigerated conditions for 9 days.	Reductions in Gram-positive and Gram-negative bacteria, suppression of LAB, reduction in fungal growth, and increased shelf life of fillets.	[117]
Sea Bass	Rosehip (*Rosa canina* L.) seed oil	The fillets were immersed in rosehip seed nanoemulsions, kept for 3 min, and subsequently stored at 4 ± 1 °C for up to 11 days.	2-day increase in microbiological shelf life in terms of TVC_m_ values.	[68]

LAB: lactic acid bacteria; TVC_m_: total viable count—mesophilic.

### 4.5. Effect of Combined Methods with EOs on Fish and Seafood Preservation

Combining EOs with other unconventional techniques represents an innovative and promising approach to improving the quality and safety of fish and seafood. Several techniques can be applied in conjunction with EOs, involving other antimicrobial agents or physical methods to preserve fish and seafood and reduce the presence of spoilage microorganisms. Among these techniques, gamma irradiation, salting, in vivo fish transport water, high hydrostatic pressure, organic acids, gamma irradiation, and ozonation stand out (Table 3).

Irradiation is an effective method for sterilizing foods, but due to the high energy input, radiation oxidizes fat and hydrolyzes amino acids, which causes foul odor and flavor [118]. Therefore, the combination with EOs may be an alternative. Lyu et al. [119] combined gamma radiation and cinnamon EO and applied them to Northern Snakehead fillets. This combination effectively inhibited microbial growth while maintaining quality and extending shelf life, and antimicrobial activity increased with radiation dose and cinnamon EO concentration. Furthermore, these combined treatments may reduce the need for higher doses of either method alone, minimizing negative effects such as lipid oxidation.

Salting is one of the oldest techniques for preserving fish, using common salt. The main cause of the preservative effect of salting is the decrease in water activity (aw). This prevents the growth of various spoilage organisms and creates a more membranous surface that further impedes the growth of microorganisms [120]. The combination of salt and oregano EO in vacuum packaging increased the shelf life of trout fillets to 16–17 days, in contrast to only 5 days for the control. Samples vacuum-packed with salt and oregano EO had lower spoilage bacteria populations than those packed with air. Sensory analysis revealed that adding salt and 0.2% oregano EO to vacuum-packed fillets resulted in a pleasant odor, increasing the acceptability of the product [121].

A practical alternative is to use EO as an anesthetic agent in sedative concentrations applied during fish transport in water. In the study by Salbego et al. [113], the effects of adding eugenol and *Lippia alba* EO to the water used to transport silver catfish (*Rhamdia quelen*) were evaluated, the results showed promising results for oxidative stress markers (TBARSs) in the liver.

However, in the experiment conducted by Cunha et al. [122], eugenol EO altered the flavor of this species’ fillets in the sensory test. For this reason, eugenol was deemed unsuitable for the anesthesia of silver catfish when the fillet was intended for human consumption.

Another technique that can be applied to fish and seafood combined with EOs is high hydrostatic pressure, a non-thermal technology for pasteurization and food preservation [123]. Kung et al. [124] reported that high hydrostatic pressure in combination with 0.2% lemon EO was more effective under the same pressure (pressures below 400 MPa) to inactivate *Morganella morganii*, a histamine-forming bacterium in fish.

The synergistic effect between EOs and chemicals such as organic acids can also be exploited to preserve fish and seafood. The combined use of tartaric acid, garlic EO, and a mixture of lactic acid and cinnamon EO showed efficacy in suppressing the degradation of fresh shrimp immersed in these solutions. These mixtures provided minimal color changes (L*, a*, and b*) and texture (hardness) throughout the storage period, being as effective as sodium metabisulfite, a traditional preservative used in shrimp [125].

In the study by Dogruyol, Mol, and Cosansu [126], the efficacy of citric acid, oregano EO, and their combination in reducing the inactivation time of *L. monocytogenes* in salmon cooked sous-vide at four different temperatures (55 °C, 57.5 °C, 60 °C, and 62.5 °C) was evaluated. All treatments significantly reduced the inactivation time compared to the control group, with the combination of citric acid and oregano EO showing greater efficacy, especially at temperatures of 57.5 °C and 60 °C. The D values, representing the time required to reduce the bacterial population by 90%, were lower in the treated groups, suggesting lower thermal resistance. Furthermore, the z values, which indicate the temperature variation required for a tenfold change in the D value, were higher in the treated groups, suggesting greater thermal sensitivity of the bacteria. These results indicate that the combination of citric acid and oregano EO can optimize heat treatment, ensuring excellent food safety by reducing the heat resistance of *L. monocytogenes*.

Gamma irradiation in combination with EOs is a promising strategy to increase the shelf life of fish and seafood. In the study by Shankar, Danneels, and Lacroix [127], the impact of coating *Merluccius* sp. fillets with a mixture of EOs and citrus extract in alginate, combined with ozonation or gamma irradiation treatments, on extending the shelf life at 4 °C was investigated. The fillets coated with the mixture of EOs in alginate and subjected to ozonation (10 ppm for 15 min) or gamma irradiation (1.0 kGy) showed promising results. While untreated fillets had a shelf life of only 7 days, gamma irradiation increased this time to more than 28 days, while ozonation extended the durability to 21 days. In addition, there were no significant changes in the pH or color of the fish during storage, indicating that their quality was maintained. These results demonstrate the effectiveness of combined techniques to optimize fish conservation.

Abdeldaiem et al. [128] also observed significant improvements in the quality of silver carp fillets treated with rosemary EO (0.5%) and subjected to irradiation (1 kGy), resulting in significantly lower counts of *S. aureus*, *Enterobacteriaceae*, and *B. cereus*. This reinforces the potential of irradiation with essential oils to ensure the microbiological safety and quality of seafood products.

Therefore, combining EOs with emerging preservative techniques has proven effective in preserving fish and seafood products. These synergistic methods increase food safety by inactivating microorganisms and offer more natural alternatives to traditional preservatives. Thus, they represent a promising solution for improving the quality and extending the shelf life of these perishable products.

**Table 3:** Application of EOs combined with other methods in fish and seafood products.

Fish and Seafood Type	Essential Oil (EO) Type	Combined Method	Application	Main Results	Reference
Shrimp (*Penaeus* spp.)	Thyme (*Thymus saturoıdes*) oil and trans-cinnamaldehyde	Gamma irradiation	Shrimps were immersed for 5 min in a coating solution with thyme EO and trans-cinnamaldehyde combined with gamma irradiation and stored at 4 °C for 21 days.	Gamma irradiation and coating treatments had synergistic effects (*p* ≤ 0.05) in reducing the APCs and *Pseudomonas putida*, with at least a 12-day shelf-life extension.	[129]
Northern Snakehead	Cinnamon (*Cinnamomum zeylanicum*) EO	Gamma irradiation	Fillets were treated with gamma irradiation and cinnamon oils and stored at 4 °C.	During storage, the combination of 1 kGy radiation and cinnamon EO displayed better inhibiting activities on aerobic plate counts, total volatile basic nitrogen, and TBARSs than 1 kGy radiation or cinnamon EO used alone.	[119]
Silver carp (*Hypophthalmichthys molitrix*)	Rosemary (*Rosmarinus officinalis*) EO	Gamma irradiation combined with EO coating	The fillets were dipped in a coating solution for 1–2 min, drained for 2 min, and dried with a gentle water flow for 30 min. The coating process was conducted twice, subsequently exposed to gamma irradiation, and stored at 4 ± 1 °C for 27 days.	Combined treatments of gamma irradiation at doses of 1, 3, and 5 kGy and edible EO coating eliminated bacteria. They extended refrigerated shelf life by up to 24 days, compared to 6 days for uncoated control samples.	[128]
Sea bream (*Sparus aurata*)	Oregano (*Origanum vulgare*) EO	Light salting and modified packaging (MAP: 40% CO_2_/30% O_2_/30% N_2_)	The fillets were salted by immersion in brine at 8 °C for 1 h, with a fish–brine ratio of 1:1 (*w*/*v*), then pipetted with oregano EO and packaged in MAP.	Adding EO to the salted MAP samples produced a distinct but pleasant flavor. It contributed to a considerably slower process of fish spoilage since the EO-treated fillets were still sensorially acceptable after 33 days of storage. The preservative effect was greater as the concentration of oregano EO was higher.	[130]
Rainbow trout (*Oncorhynchus mykiss*)	Oregano (*Origanum vulgare*) EO	Salting and vacuum packaging	The fillets were salted by immersion in brine, with a fish–brine ratio of 1:1 and brine temperature of 8 °C, for 1 h and placed in packages and subsequently applied to the same oregano EO using a micropipette; then, massaged for uniform distribution and subsequently kept in vacuum refrigeration (4 ± 0.5 °C) for 18 days.	The addition of salt extended the product’s shelf life by 9 days, while the combination of salt and oregano EO under vacuum conditions resulted in a significant extension of the shelf life of trout fillets, by approximately 11–12 days.	[121]
Silver catfish (*Rhamdia quelen*)	*Lippia alba* (Mill.) NE Brown (Verbenaceae) EO	Fish transport water in vivo	In vivo fish were exposed to water during transport to the cold storage facility with *Lippia alba* EO and kept on frozen storage after slaughter.	EO delayed the peak formation of peroxides (from the third to the sixth month of storage) and of TBARSs (from the ninth to the twelfth month of storage) compared to the control fillets.	[131]
Silver catfish (*Rhamdia quelen*)	*Aloysia triphylla* (L’Her.) EO	Fish transport water in vivo	The fish, in vivo, were exposed to water containing *A. triphylla* EO for 6 h while being transported to the refrigerator, and after slaughter, they were kept under refrigeration (2 ± 1 °C) for 35 days.	During transportation, silver catfish treated with *A. triphylla* EO had lower sensory demerit scores after 10 days of ice storage, indicating improved fish quality and freshness.	[132]
Rainbow trout (*Oncorhynchus mykiss*)	Clove (*Syzygium aromaticum*) EO	High-pressure processing (HPP) combined with chitosan film	Fillets were treated with high pressure for 10 min at 300 Mpa at 12 °C and stored at 4 °C for 22 days.	A significant additive effect was observed between films and high-pressure treatments, inhibiting aerobic bacteria and mesophilic coliforms.	[133]
Tiger shrimps (*Penaeus monodon*)	Treatments in solutions of EOs ((cinnamon (*Cinnamomum zeylanicum*), garlic oil (*Allium sativum*), and lemon oil (*Citrus aurantifolia*)) and organic acids (lactic acid, tartaric acid, and sodium diacetate)	Organic acids	Shrimps were dipped in solutions of EOs (cinnamon oil, garlic oil, and lemon oil) and organic acids (lactic acid, tartaric acid, and sodium diacetate) for 30 min. Shrimps were drip-dried for 5 min, packed in labeled polyethylene containers, and stored in a refrigerator (4 °C) for 10 days.	Mixtures of tartaric acid, garlic oil, lactic acid, and cinnamon oil suppressed spoilage of fresh shrimp.	[125]

APC: aerobic plate count; TBARSs: thiobarbituric acid-reactive substances.

## 5. Effect of EOs on the Production of Biogenic Amines in Fish and Seafood

Biogenic amines (BAs) in fish have been implicated as an important causative agent of foodborne diseases, where intoxication results from the ingestion of foods containing higher amounts of biogenic amines, so their levels are important to characterize the freshness and hygienic quality of aquatic products [134].

BAs are biologically active, have low molecular weights, and contain nitrogen-containing organic bases. They are formed by microbial decarboxylation of amino acids or amination and transamination of aldehydes or ketones [135]. Removal of the α-carboxyl groups from protein amino acids produces the corresponding BAs.

**Figure 4:**
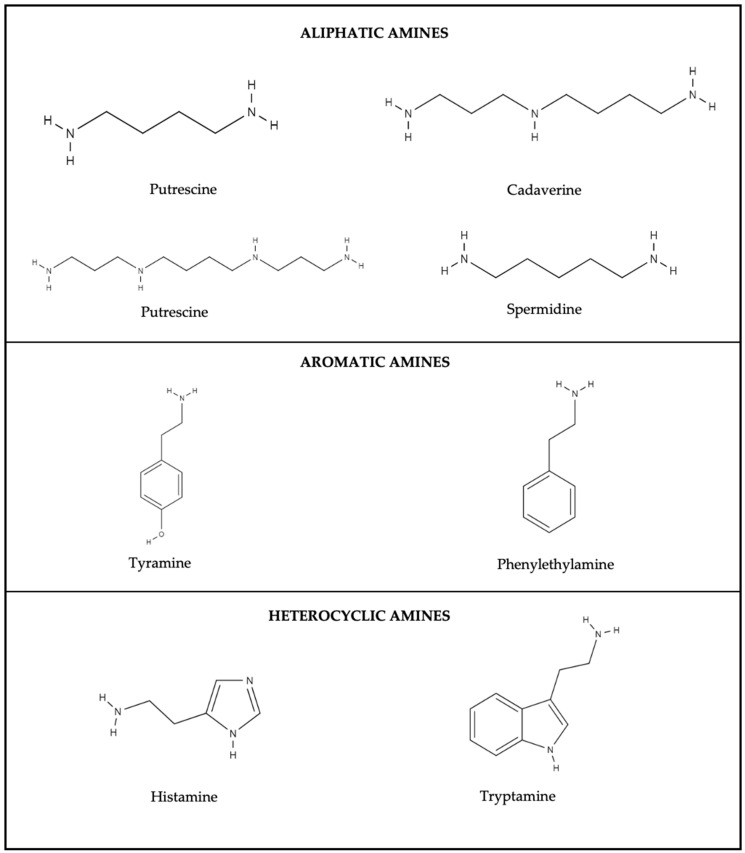
Chemical structures of some important biogenics in fish and seafood products.

According to the structure, BAs can be aliphatic (putrescine, cadaverine, spermine and spermidine), aromatic (phenylethylamine and tyramine), and heterocyclic (histamine and tryptamine) (Figure 4), generated mainly by the decarboxylation of their corresponding precursor amino acids [136]. They are produced during fish processing and storage as a result of inadequate storage conditions and microbial contamination by microorganisms with decarboxylase enzymatic activity that convert amino acids into their respective biogenic amines [137].

Some studies have shown that using EOs can significantly extend the shelf life of fish by reducing the formation of biogenic amines, thus maintaining better quality for a more extended period. Křížek et al. [138] investigated the effects of EO treatments (thyme and oregano) and UV-C irradiation (doses of 121 and 243 mJ/cm^2^) on the formation of biogenic amines in vacuum-packed carp (*Cyprinus carpio*) fillets. Sensory signs of spoilage in the fillets appeared on the 9th day in the control samples, on the 14th day in the UV-treated samples, and on the 35th and 42nd day for the thyme and oregano EOs, respectively. Oregano EO was the most effective in suppressing putrescine, cadaverine, tyramine, and phenylethylamine, while UV-C irradiation was less effective. The EO-treated samples maintained good quality, with less than 10 mg/kg of biogenic amines, and showed a positive impact on sensory properties, increasing the shelf life of carp fillets by 5 to 6 times. The polyamines spermidine and spermine showed no significant changes, and tryptamine was not detected in the EO-treated samples.

Histamine and tyramine are the most important BAs in fish and fishery products [139]. Histamine poisoning from fish is a foodborne chemical, also known as scombroid. It is considered an acute disease, being the most common cause of ichthyotoxicosis worldwide. It results from ingesting fish with high levels of accumulated histamine or other biogenic amines. This occurs because certain bacteria produce the enzyme histidine decarboxylase during growth. This enzyme reacts with histidine, a natural amino acid that is present in higher amounts in some fish than in others. The result is the formation of scombroid toxin (histamine), which can accumulate. This occurs most commonly when fish are kept at ambient or high temperatures (21.1 °C–32.2 °C) for several hours, but can occur at more moderate temperatures (≥7.2 °C). There is no unified international standard for BAs in food; however, the FDA has set a suggested allowable limit of 50 mg/kg of histamine [140].

The types of bacteria associated with histamine production are commonly present in the saltwater environment, and the main bacteria responsible for histidine decarboxylation and histamine toxicity in fish are members of the family Enterobacteriaceae [141]. They occur naturally on the gills, external surfaces, and intestines of live saltwater fish without causing harm to the fish. After death, the fish’s defense mechanisms no longer inhibit bacterial growth in muscle tissue, and histamine-forming bacteria may begin to grow, resulting in histamine production. Gutting and removing the gills can reduce, but not eliminate, the number of histamine-forming bacteria. Packing the visceral cavity with ice can aid in cooling large fish in which internal muscle temperatures are not easily reduced. However, when performed improperly, these steps can accelerate histamine development in the edible portions of the fish, spreading the bacteria from the visceral cavity to the whole fish [142].

Therefore, interventions with EOs may be an alternative for bacterial control by inhibiting the growth of bacteria with histidine decarboxylase activity. In the study by Cai et al. [98], red drum (*Sciaenops ocellatus*) fillets treated with 4 mL/L of clove, cumin, and mint EOs inhibited microbiological properties throughout the storage periods. All treatments were effective in delaying the sensory deterioration of the fish, exhibiting a positive effect and resulting in low levels of biogenic amines, mainly histamine, putrescine, and cadaverine. Similarly, treatment with 0.1% cinnamon bark oil improved sensory quality, inhibited microbial growth, and delayed increases in TVB-N, putrescine, cadaverine, and the K value in grass carp fillets [92].

Another problem linked to fish quality is related to oxidative stress. Fish fillets have a high propensity for oxidative processes during frozen storage due to the significant presence of polyunsaturated fatty acids [143]. This undesirable phenomenon culminates in generating unpleasant flavors and aromas, which restrict the shelf life of the fish [144]. Some EOs play an important role in reducing oxidative stress in fish fillets; studies on these EOs are still embryonic but very promising. In the work of Hu et al. [145], the effect of a collagen film containing cinnamon, oregano, and clove EOs on the quality of mackerel fillets during storage was evaluated. The results showed that the samples treated with the EO-containing film had lower levels of TVB-N, substances related to thiobarbituric acid, and pH, indicating a reduction in deterioration and oxidation of the fillets. Furthermore, the EOs exhibited antibacterial activity, with cinnamon oil showing the greatest efficacy due to cinnamaldehyde. The sensory scores also indicated a prolonged acceptability of the treated samples.

In a study on lipid oxidative stability in shrimp hepatopancreas with EOs, it was found that lemon EO (200 ppm) delayed the increase in thiobarbituric acid reactive substances (TBARSs) and the r-anisidine value (AnV) of lipids during storage, and consequently decreased the formation of volatile compounds and a rancid odor [89]. Volpe et al. [146] investigated a combination of methods to evaluate the efficacy of edible carrageenan coatings, with or without the addition of lemon EO, to delay the deterioration of muscle structure, lipid peroxidation, and olfactory characteristics of rainbow trout fillets stored at 4 °C for up to 12 days. The lemon EO-incorporated coating delayed the development of lipid peroxidation and TBARS values more slowly than the uncoated samples during storage. Furthermore, electronic nose analysis revealed that the carrageenan coating incorporating EO better preserved the olfactory characteristics of the trout fillets compared to the carrageenan coating alone. The EO-enriched carrageenan coating best preserved the fresh trout fillet’s morphological, physicochemical, and olfactory characteristics.

Essential oils have demonstrated significant potential in fishery products as oxidative retarders and biogenic amine reducers. Furthermore, essential oils exhibit antioxidant properties that help maintain low TBARS values, indicating reduced lipid oxidation [147]. Incorporation of essential oils into fish coatings not only inhibits microbial growth, thereby reducing biogenic amine accumulation but also increases the oxidative stability of fish fillets, as seen with lower TBARS values in treated samples compared to controls [148]. Overall, applying essential oils in fish preservation effectively addresses oxidative spoilage and safety concerns associated with biogenic amines [149].

## 6. Challenges and Future Perspectives in the Use of EOs in Fish and Seafood

Consumer acceptance of using EOs in fish and seafood is a critical factor for their application in preserving these foods. Although EOs provide significant functional benefits, such as antimicrobial and antioxidant properties, their sensory impact can limit acceptance, primarily due to low palatability residues or intense aromas. This sensory impact varies depending on the application method, exposure time, type of EO, and concentrations. For example, studies have shown that applying thyme and oregano EOs on carp fillets improved sensory properties, suggesting a positive effect when used appropriately [138].

However, the immersion of raw catfish fillets in solutions containing carvacrol—a common component in oils like thyme and oregano—resulted in negative sensory alterations, likely due to high concentrations or improper application methods [72]. This duality highlights the need to balance functional efficacy and sensory acceptance. Strategies such as using nanoemulsions, which enable controlled release and reduce sensory impact, have shown promise in overcoming these challenges. Another alternative is combining EOs with other preservatives or technologies, reducing the required concentrations to achieve the desired activity. Additionally, selecting EOs with milder sensory profiles or developing encapsulation techniques could help minimize negative impacts, broadening their applicability to a wide range of products, including those with delicate sensory profiles. These approaches, aligned with consumer perception, can enhance the commercial viability of EOs as natural preservatives.

Few studies have compared the different methods of applying essential oils (EOs) to fish and seafood, an area that warrants further exploration. Research comparing these methods has revealed significant differences in outcomes depending on the technique used. For instance, a study by Navarro-Segura et al. [150] found that vacuum vapor treatment with oregano EO more effectively extended the shelf life of farmed, refrigerated fish fillets, lasting at least 28 days at 4 °C. In contrast, the conventional immersion method only extended the shelf life to 12 to 14 days. 

The seafood industry should invest in offering fish and seafood with extended shelf life, using this feature as a competitive differentiator to attract consumers. This increased product durability can justify the higher costs of using essential oils, offsetting the initial investments. Additionally, the seafood industry can adopt a sustainable approach by promoting natural preservatives and obtaining certifications or seals that reinforce this commitment.

This strategy not only adds value to the products but also caters to a growing audience of consumers concerned with health, sustainability, and reducing chemical additives in food. By communicating the benefits of essential oils, such as their antimicrobial and antioxidant efficacy, and associating their products with sustainable practices, industries can build a distinctive brand image and strengthen their position in the market. This combination of technological innovation, sustainability, and marketing can be a powerful tool to increase acceptance and demand for fish and seafood preserved with EOs.

## 7. Conclusions

The use of EOs in fish and seafood preservation as an alternative solution to combat microorganisms and extend the shelf life of these products was reviewed. The antimicrobial properties of EOs are influenced by their chemical composition, which varies according to the conditions of cultivation, processing, and storage. The ability of EOs to function as antimicrobials is influenced by their chemical and physical properties.

The main methods of application of EOs include immersion, spraying, and pipetting, and their antimicrobial efficacy is influenced by the concentration, exposure time, and food characteristics, such as chemical composition and biofilms. To overcome the limitations of direct application, immersion of fish and seafood in solutions containing EOs nanoemulsions has been shown to improve sensory quality and extend shelf life significantly. Furthermore, EOs can be combined with other agents, such as bacteriocins, in edible coatings, offering a sustainable and effective solution for preserving these foods. This approach extends the shelf life of products and improves their sensory properties, minimizing the strong aroma of EOs.

Combining EOs with other unconventional techniques appears to be an innovative and promising approach to improving the quality and safety of fish and seafood. The combined use of EOs with antimicrobial agents or with gamma irradiation, salting, high hydrostatic pressure, organic acids, and ozonation has demonstrated effectiveness in preserving these foods and reducing spoilage microorganisms, increasing their shelf life and safety for consumption.

Future perspectives include investigating ways to optimize EO concentrations to minimize negative sensory impacts and exploring synergies with other preservation techniques, such as applying functional nanocomposite films and developing advanced nanoemulsions to meet the growing demand for natural and safe food products. 

Discoveries and innovations will likely emerge as research and development advances. These discoveries and innovations will further advance knowledge about fish preservation with EOs. To ensure that preservation practices are effective, safe, sustainable, and accepted by the market, scientists, industry, regulators, and consumers must work together.

## Data Availability

No new data were created or analyzed in this study. Data sharing is not applicable to this article.
